# Correction: Alternative splicing expands the antiviral IFITM repertoire in Chinese rufous horseshoe bats

**DOI:** 10.1371/journal.ppat.1014001

**Published:** 2026-02-25

**Authors:** Nelly S. C. Mak, Jingyan Liu, Dan Zhang, Jordan Taylor, Xiaomeng Li, Kazi Rahman, Feiyu Chen, Siddhartha A. K. Datta, Kin Kui Lai, Zhengli Shi, Nigel Temperton, Aaron T. Irving, Alex A. Compton, Richard D. Sloan

In the Funding section, the grant number from the funder National Science Foundation of Zhejiang Province is listed incorrectly. The correct grant number is: Z23C010001.
