# Supplementary figures and images for: Alternative splicing expands the antiviral IFITM repertoire in Chinese rufous horseshoe bats
Source: PLoS Pathog. 2024 Dec 26;20(12):e1012763. doi: 10.1371/journal.ppat.1012763 (PMC11801718; doi:10.1371/journal.ppat.1012763)

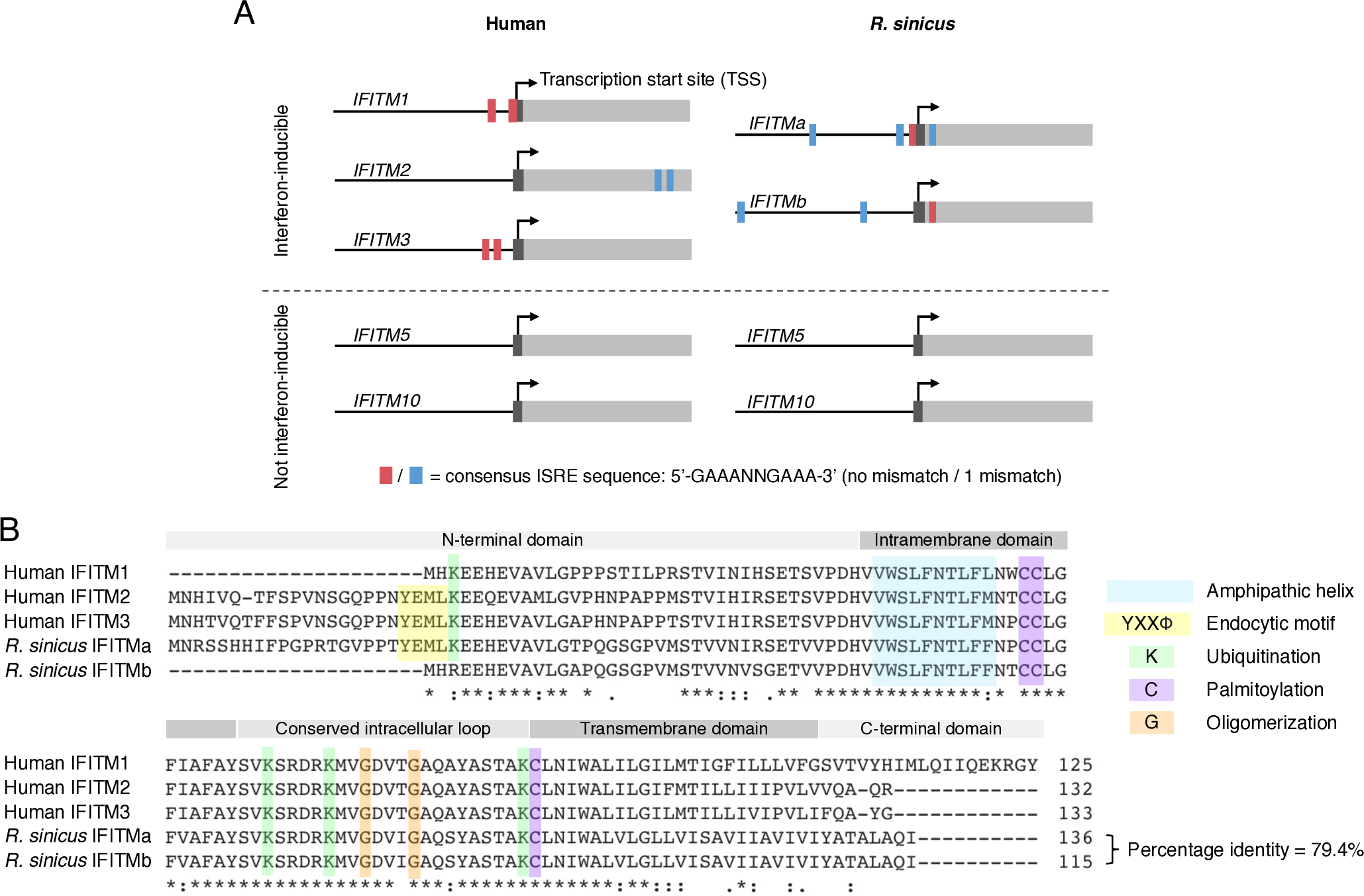

Supplement: S1 Fig — A. Identification of interferon-stimulated response elements (ISRE) around the transcription start site (± 350 base pairs) of human and R. sinicus IFITM genes. ISREs were defined to have the consensus sequence of GAAANNGAAA or TTTCNNTTTC, with no mismatch (red) or 1 mismatch (blue) [104]. B. Amino acid sequence alignment of human IFITM1-3 and R. sinicus IFITMs. Protein domains, functional motifs and amino acids that undergo post-translational modifications are highlighted. Asterisks (*) indicate positions with a conserved residue; colons (:) and periods (.) indicate conservation between groups of strongly and weakly similar properties respectively. Percentage identity was calculated using the pairwise sequence alignment tool EMBOSS Needle [84]. (TIF) [file ppat.1012763.s001.tif]

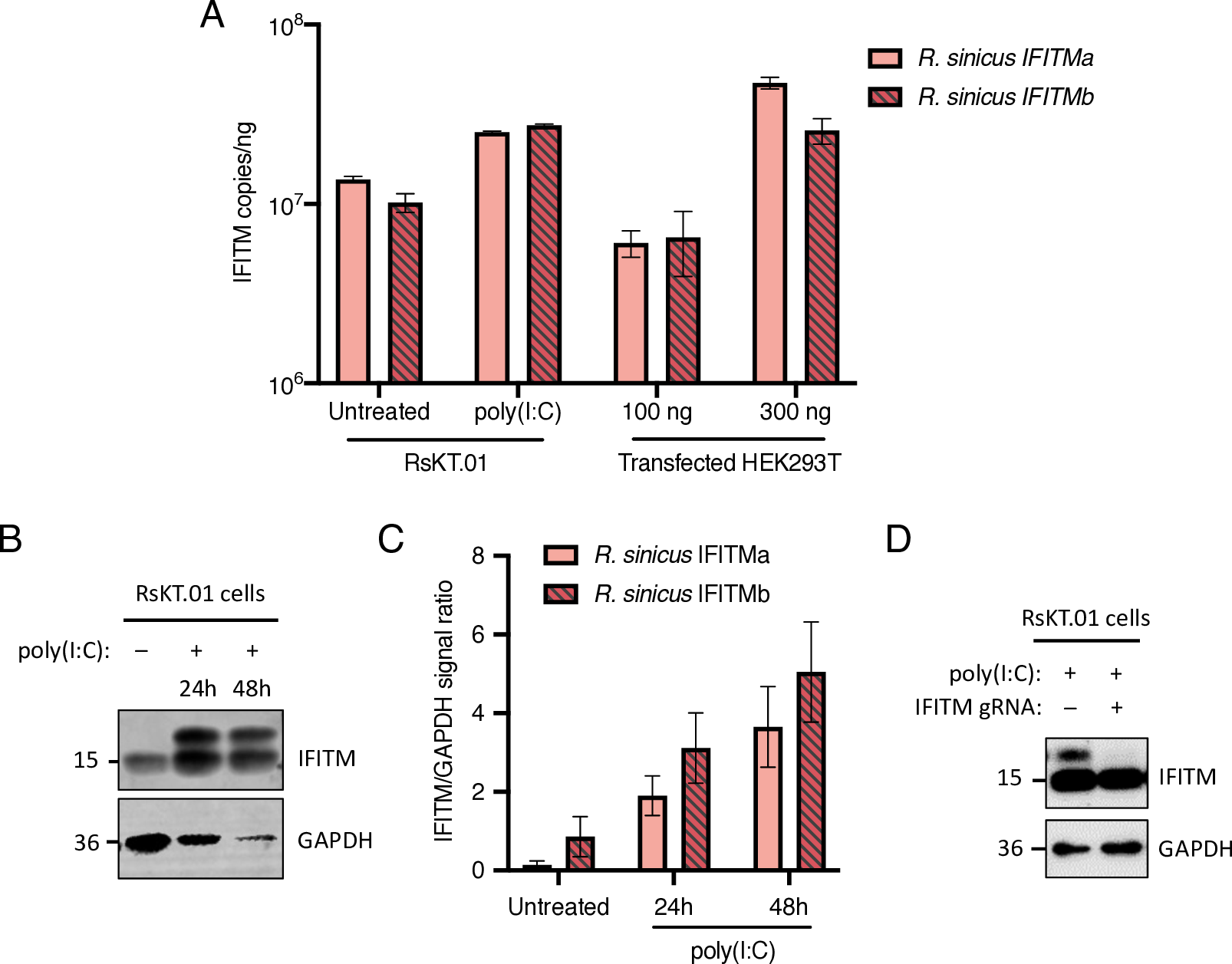

Supplement: S2 Fig — A. Absolute quantification of IFITM mRNA abundance was performed by RT-qPCR. Untreated and poly(I:C)-treated RsKT.01 cells, and HEK293T transfected with R. sinicus IFITMa or IFITMb constructs, were subjected to RNA extraction and RT-qPCR with isoform-specific primers. Exact copy numbers per ng of RNA were determined by normalizing Cq values against a standard curve. Error bars represent SEM of averages from 3 independent experiments, each performed in triplicate. B. RsKT.01 cells were untreated or transfected with poly(I:C), followed by detection of IFITM expression by western blotting at 24 and 48 hours post-transfection. Representative western blot from 3 independent experiments is shown. C. Quantitative analysis of the IFITM/GAPDH ratio from western blots. Error bars represent SEM of averages from 3 independent experiments. D. Knockout cells were generated by CRISPR/Cas9-mediated knockout using a guide RNA that targets the first exon of rsIFITMa. Wild-type cells and rsIFITMa knockout cells were transfected with poly(I:C), followed by detection of IFITM expression by western blotting at 48 hours post-transfection. (TIF) [file ppat.1012763.s002.tif]

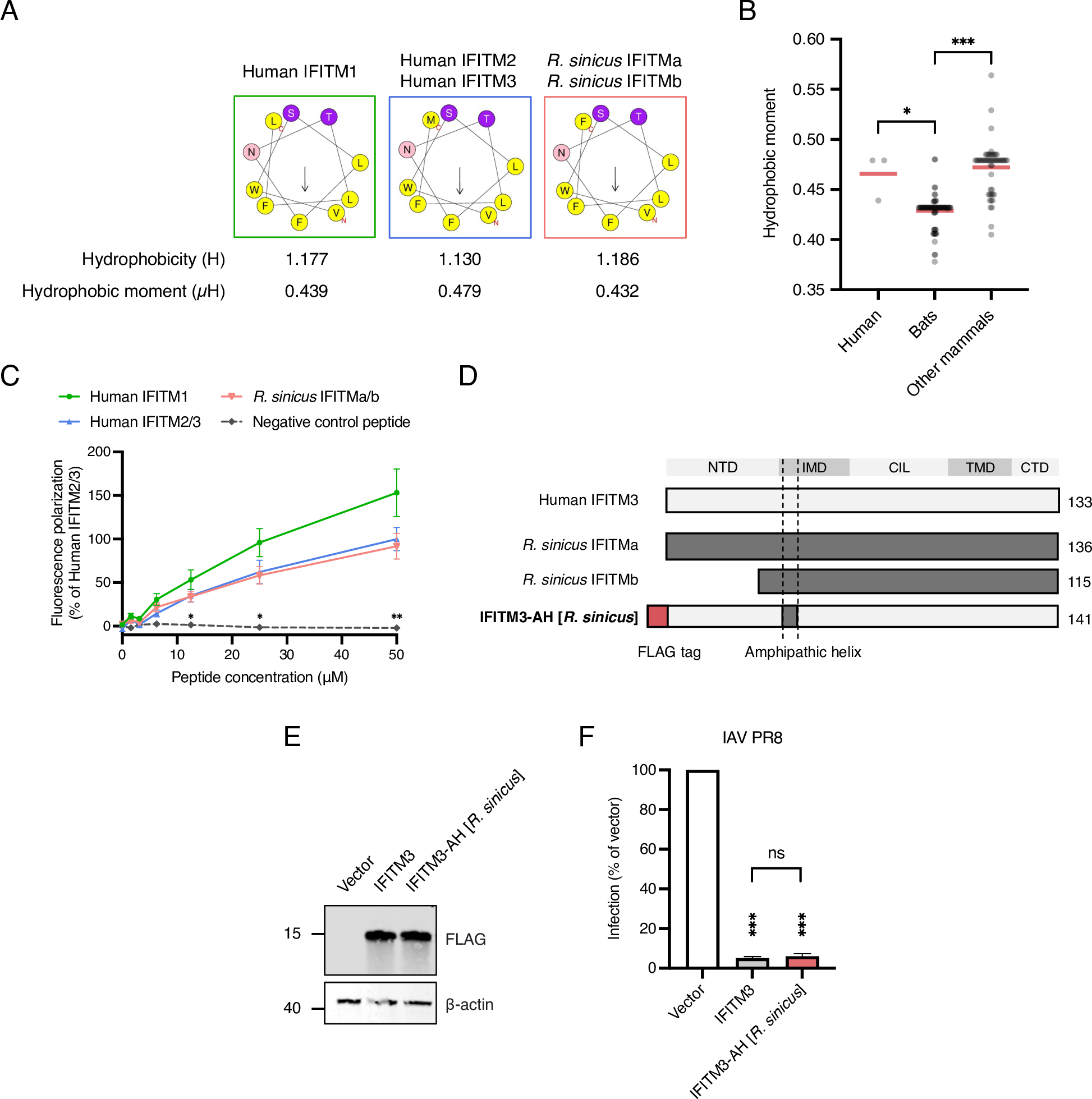

Supplement: S3 Fig — A. Helical wheel project plots of human and R. sinicus IFITM amphipathic helices containing hydrophobic (yellow) and hydrophilic (purple or pink) residues. Arrows indicate magnitude and direction of mean hydrophobic moments. B. Mean hydrophobic moment of IFITM amphipathic helices from 34 mammalian species, including 19 bat species. Medians of each group are shown. Kruskal-Wallis test; *p<0.05, ***p<0.001. C. NBD-cholesterol fluorescence polarization was measured following incubation of IFITM amphipathic helix peptides (0–50 μM) with NBD-cholesterol (500 nM). Data points are normalized to 50 uM human IFITM2/3. Error bars represent SEM of averages from 3 independent experiments. Statistical significance of differences between human IFITM2/3 and another peptide was determined by one-way ANOVA with Dunnett’s test; *p<0.05, **p<0.01. D-F. HEK293T cells were transfected with FLAG-tagged IFITM3 or chimeric IFITM3-AH [R. sinicus] as illustrated in (D), and protein expression was detected by western blotting at 24 hours post-transfection (E). Transfected cells were infected with IAV at MOI = 0.05 and analyzed by flow cytometry at 18 hours post-infection. Error bars represent SEM of averages from 3 independent experiments, each performed in duplicate. Statistical significance of difference between vector- and IFITM-transfected cells was determined by one-way ANOVA with Dunnett’s test; Statistical significance of difference between IFITM3- and IFITM3-AH [R. sinicus]-transfected cells was determined by unpaired t-test; ***p<0.001; ns, non-significant. (TIF) [file ppat.1012763.s003.tif]

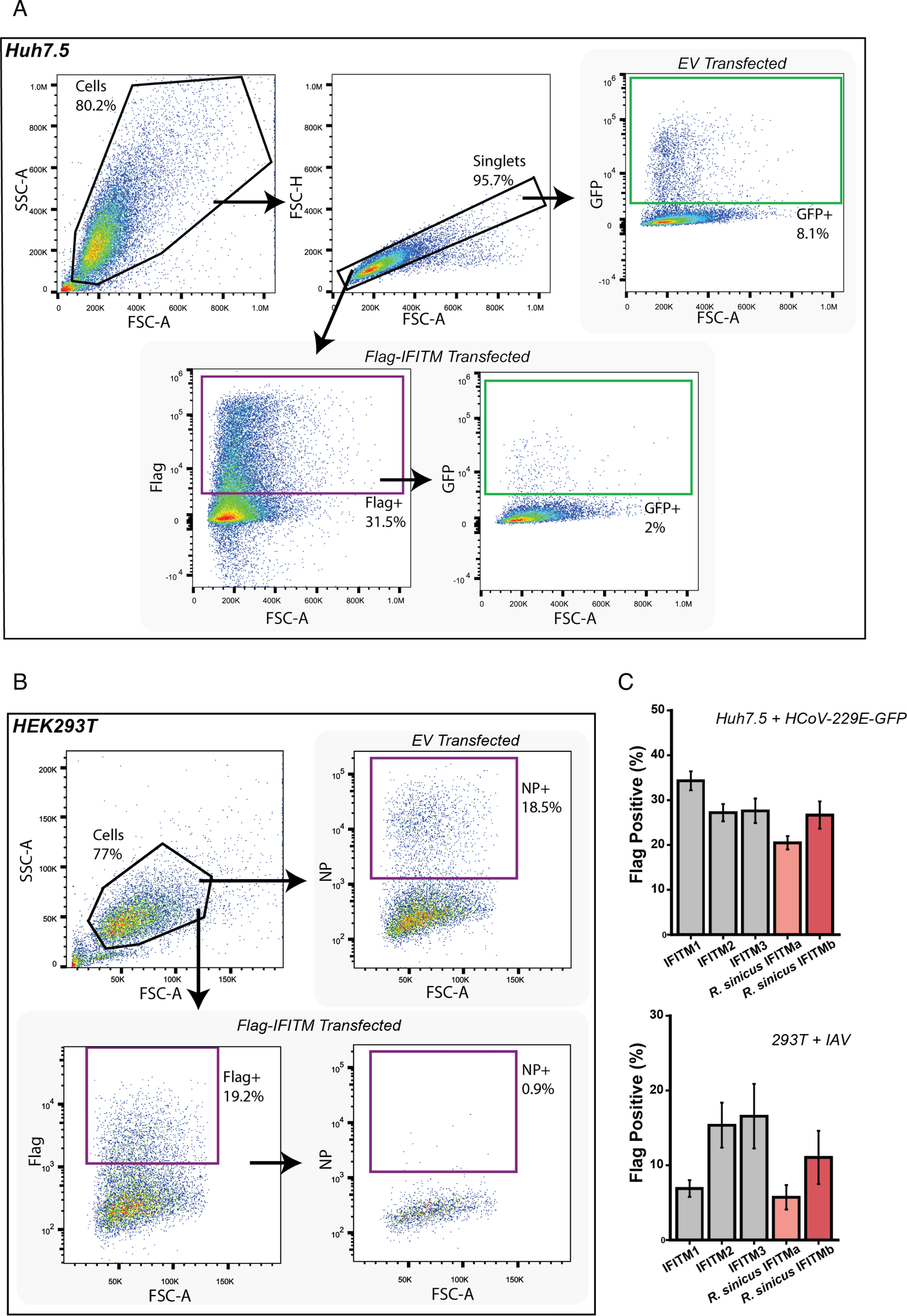

Supplement: S4 Fig — A-B. Huh7.5 (A) or HEK293T (B) cells were transfected with FLAG-tagged IFITM constructs and infected with HCoV-229E-GFP or IAV for 18 hours respectively. Flow cytometry dot plots show the gating strategy to identify cells that are double positive for FLAG staining and GFP or IAV nucleoprotein (NP) staining. Percentages of gated cells within the parent population are shown. C. Percentage of successfully transfected cells were determined by gating for FLAG. Error bars represent SEM of averages from 3 independent experiments. EV, empty vector. (TIF) [file ppat.1012763.s004.tif]

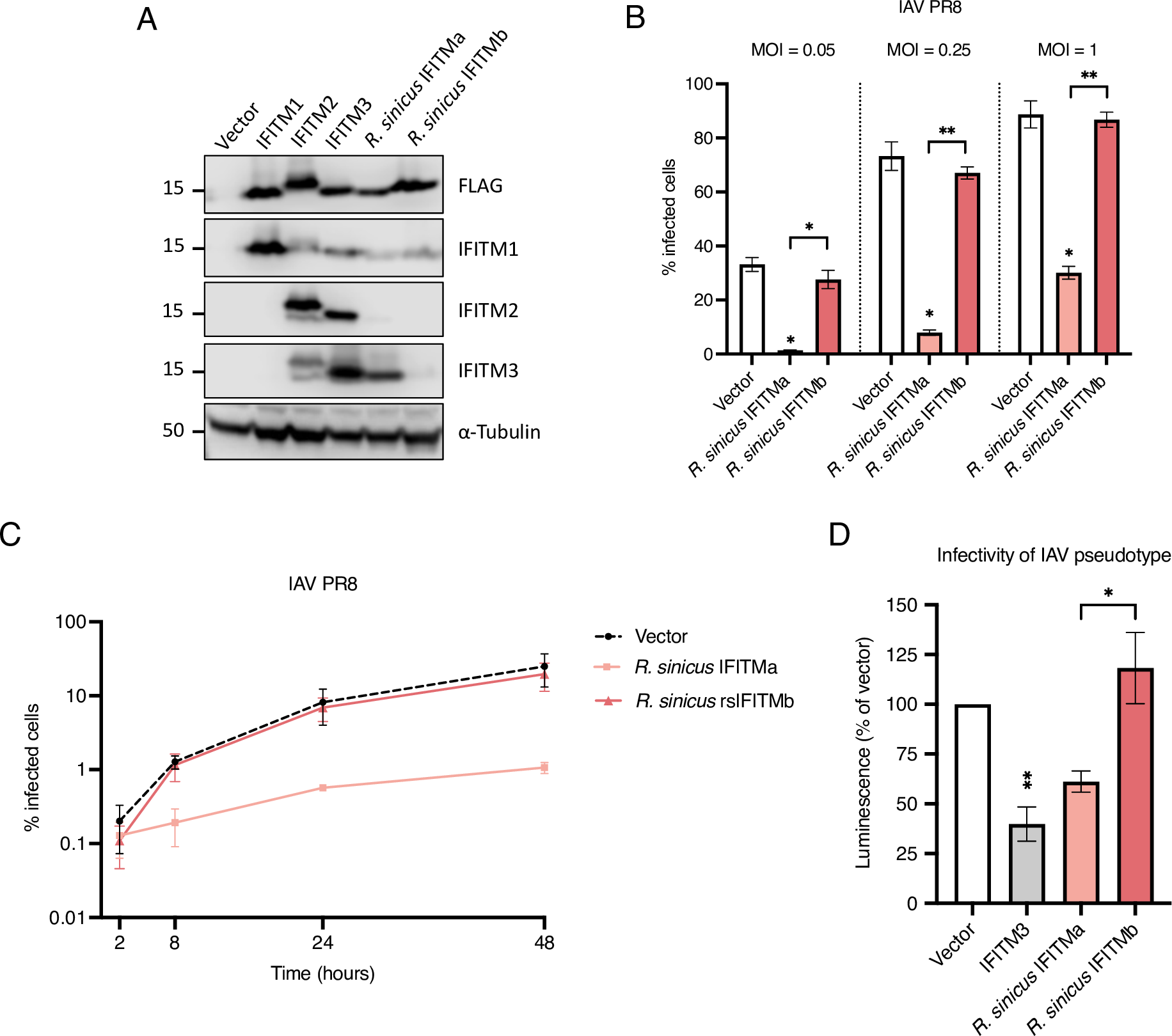

Supplement: S5 Fig — A. HEK293T cells were stably transduced to express the indicated IFITMs. IFITM expression was detected by western blotting. B. Stably transduced HEK293T cells were infected with IAV at the indicated MOIs and analyzed for NP staining by flow cytometry at 18 hours post-infection. Error bars represent SEM of averages from 3 independent experiments, each performed in duplicate. C. Stably transduced HEK293T cells were infected with IAV at MOI = 0.05 and analyzed for NP staining by flow cytometry at the indicated time points. Error bars represent SEM of averages from 3 independent experiments, each performed in duplicate. D. IAV pseudotypes encoding a luciferase reporter were produced from HEK293T cells stably expressing the indicated IFITMs. RT-normalized IAV pseudotypes were used to transduce wild-type HEK293T cells to determine infectivity. Cells were lysed and analyzed by luciferase assay after 48 hours. Error bars represent SEM of averages from 3 independent experiments. Statistical significance of difference between vector- and IFITM-expressing cells was determined by one-way ANOVA with Dunnett’s test; statistical significance of difference between R. sinicus IFITMa- and IFITMb-expressing cells was determined by unpaired t-test; *p<0.05, **p<0.01. (TIF) [file ppat.1012763.s005.tif]

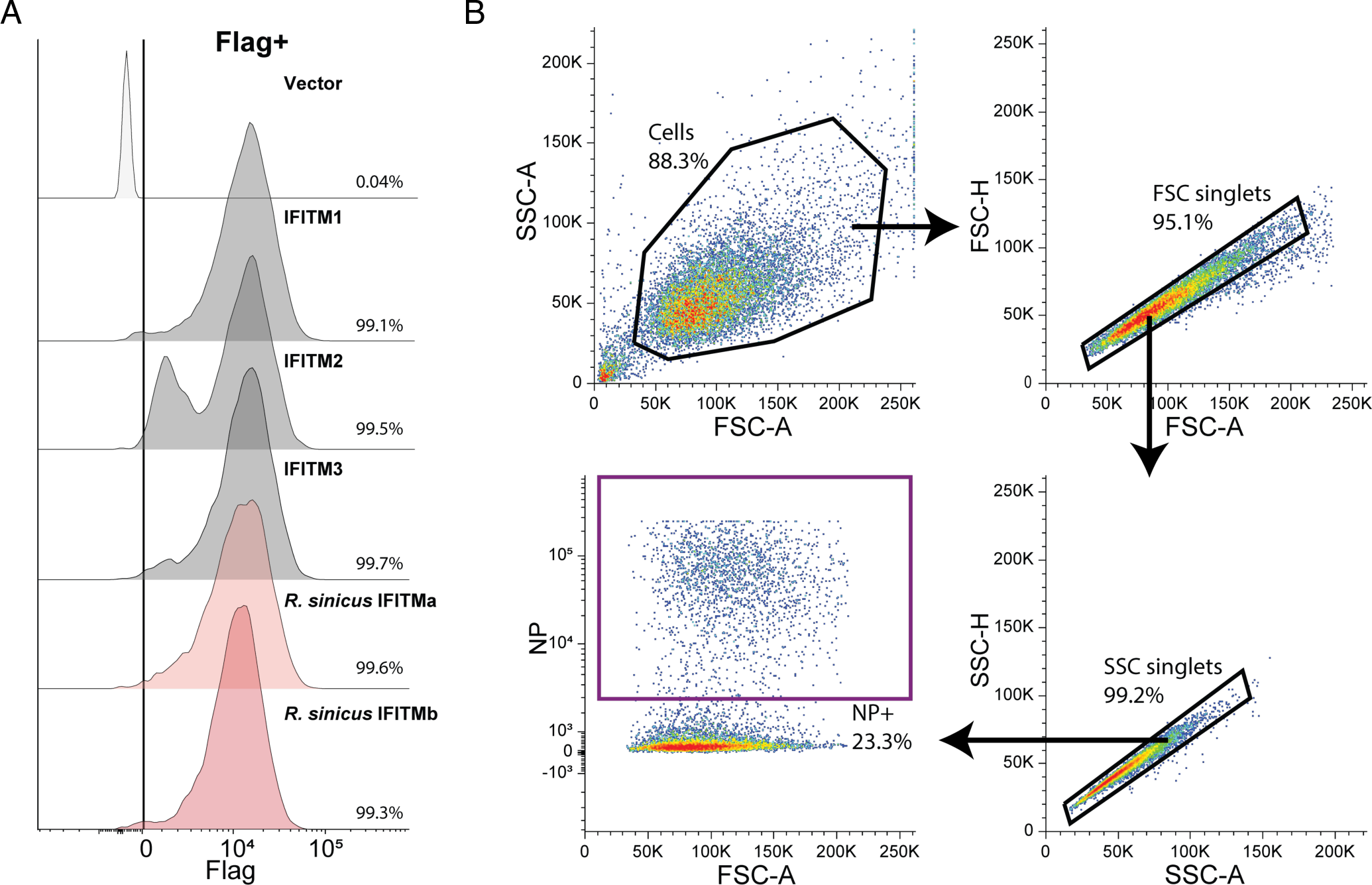

Supplement: S6 Fig — A. HEK293T cells were stably transduced to express the indicated FLAG-tagged IFITMs. Histograms show IFITM expression assessed by FLAG staining and the percentage of FLAG-positive cells. B. HEK293T cells stable expressing FLAG-tagged IFITMs were infected with IAV. Flow cytometry dot plots show the gating strategy to identify cells that are positive for IAV NP staining. Percentages of gated cells within the parent population are shown. (TIF) [file ppat.1012763.s006.tif]

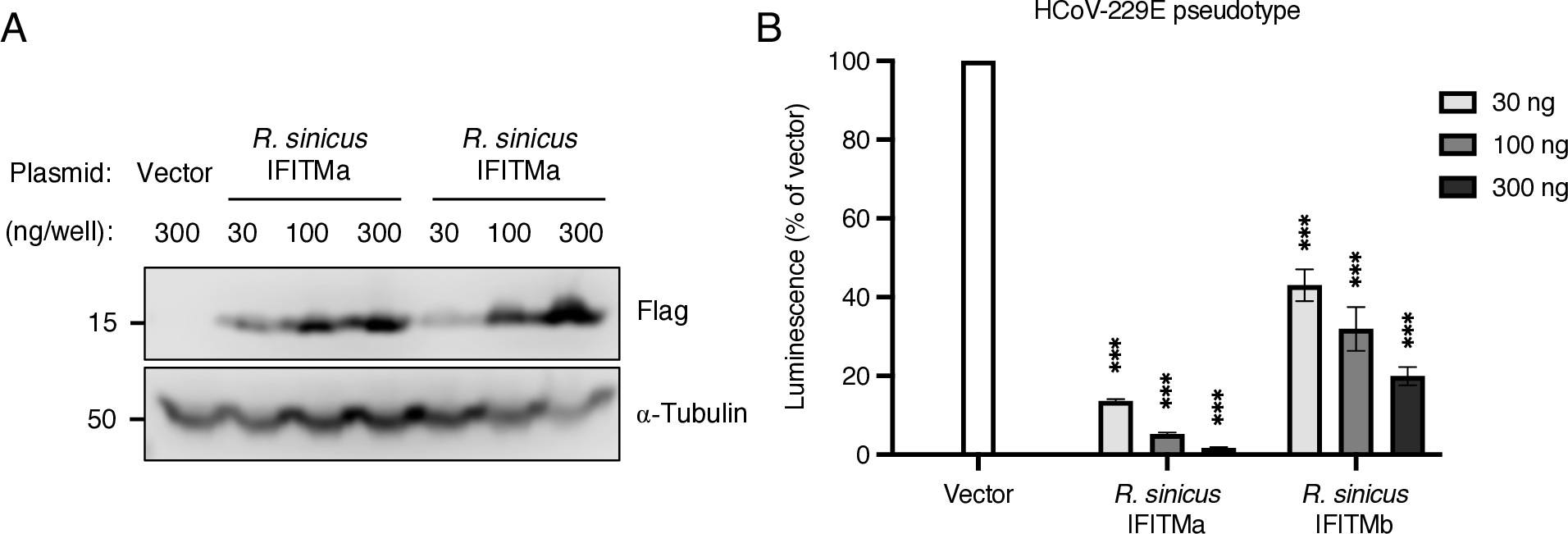

Supplement: S7 Fig — A. HEK293T cells were transfected with the indicated amount of FLAG-tagged R. sinicus IFITMa or IFITMb in 24-well plates. The total amount of transfected DNA was kept constant with an empty plasmid. IFITM expression was determined by western blotting at 24 hours post-transfection. B. HEK293T cells were co-transfected with the indicated amount of rsIFITMa or rsIFITMb and APN then transduced with HCoV-229E pseudotypes encoding a luciferase reporter. Cells were lysed and analyzed by luciferase assay after 48 hours. Error bars represent SEM of averages from 3 independent experiments, each performed in triplicate. Statistical significance of difference between vector- and IFITM-expressing cells were determined by one-way ANOVA with Dunnett’s test; ***p<0.001. (TIF) [file ppat.1012763.s007.tif]

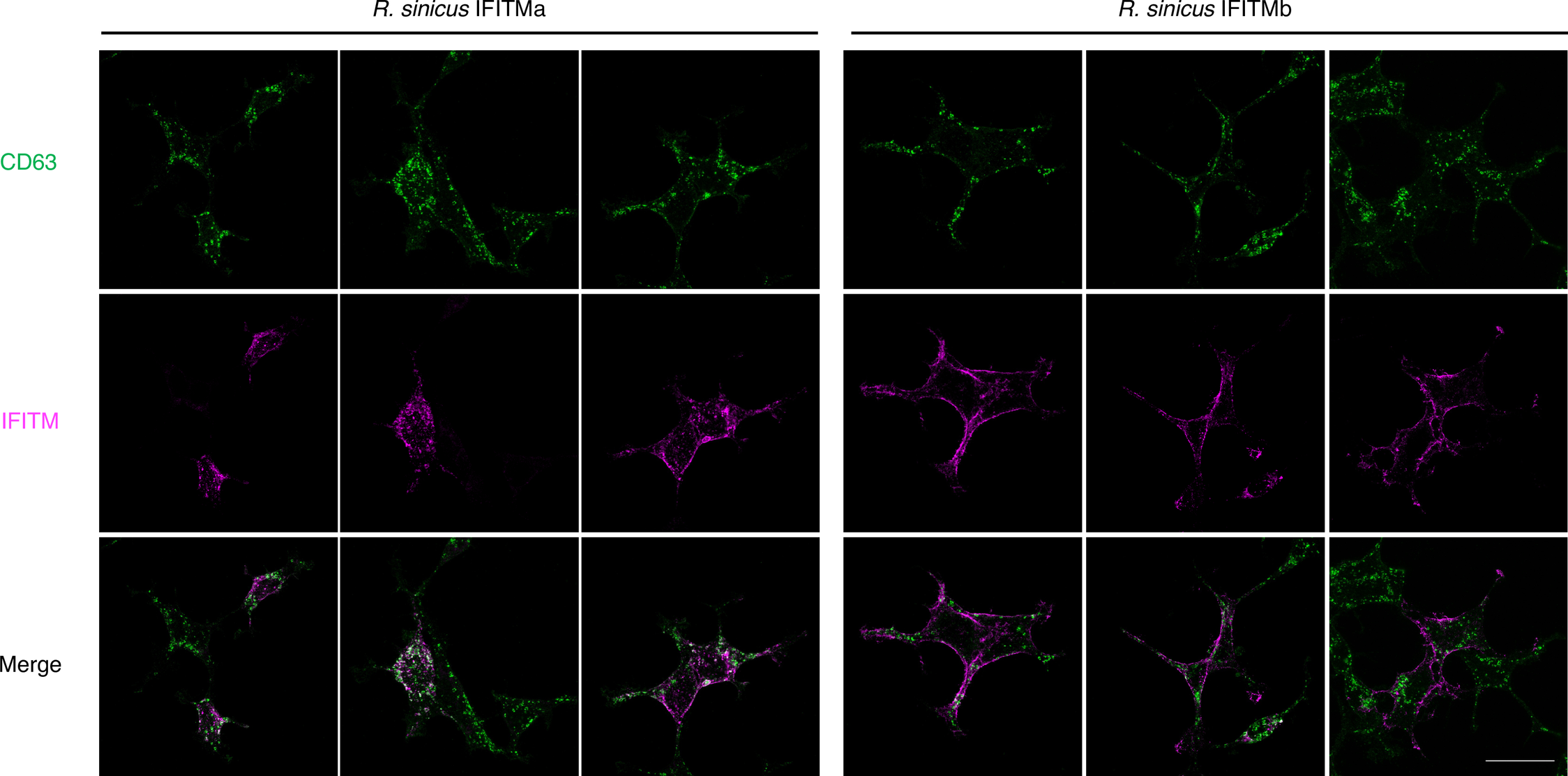

Supplement: S8 Fig — HEK293T cells were transfected with FLAG-tagged R. sinicus IFITMa or IFITMb. Cells were stained for CD63 (green; late endosome marker) and FLAG (magenta; IFITMs) at 48 hours post-transfection and imaged by confocal microscopy. Representative z-stack images are shown. Scale bar, 30μm. (TIF) [file ppat.1012763.s008.tif]

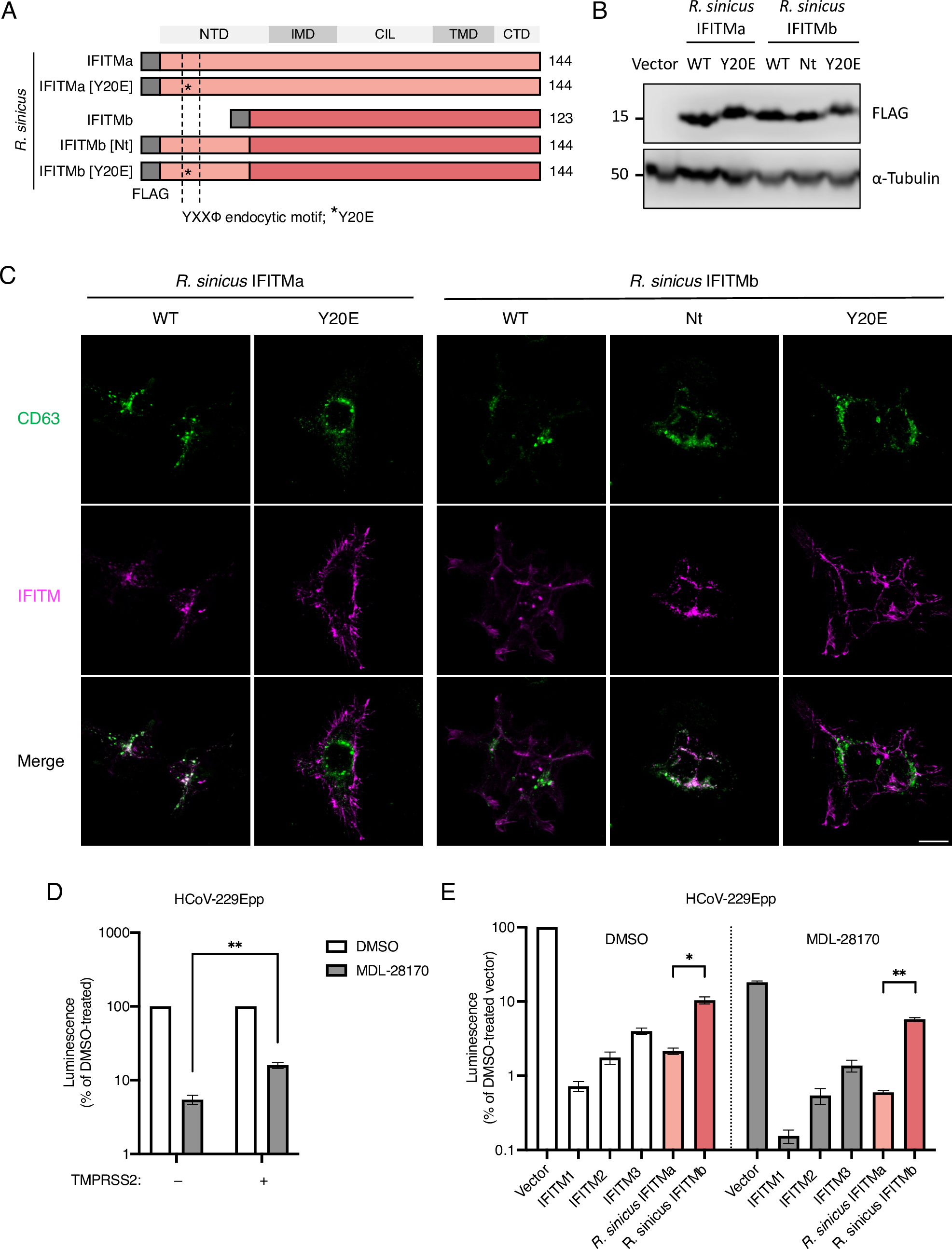

Supplement: S9 Fig — A. Schematic showing R. sinicus IFITMa and IFITMb N-terminal mutants. B. HEK293T cells were transfected with FLAG-tagged R. sinicus IFITMs with the indicated mutations. IFITM expression was detected by western blotting at 24 hours post-transfection. C. HEK293T cells were transfected with the indicated FLAG-tagged IFITMs. Cells were stained for CD63 (green; late endosome marker) and FLAG (magenta; IFITMs) at 48 hours post-transfection and imaged by confocal microscopy. Representative z-stack images are shown. Scale bar, 15 μm. D. HEK293T and HEK293T-ACE2-TMPRSS2 cells were transfected with APN and transduced with HCoV-229E pseudotypes in the presence of DMSO or MDL-28170. Cells were lysed and analyzed by luciferase assay after 48 hours. Data points were normalized to the respective DMSO-treated cells (white bars). Error bars represent SEM of averages of 3 independent experiments, each performed in triplicate. E. HEK293T-ACE2-TMPRSS2 cells were co-transfected with APN and the indicated FLAG-tagged IFITM constructs, then transduced with HCoV-229E pseudotypes in the presence of DMSO or MDL-28170. Cells were lysed and analyzed by luciferase assay after 48 hours. Data points were normalized to vector/DMSO. Error bars represent SEM of averages from 3 independent experiments, each performed in triplicate. Statistical significance of difference between indicated groups were determined by unpaired t-test; *p<0.05, **p<0.01, ***p<0.001. (TIF) [file ppat.1012763.s009.tif]

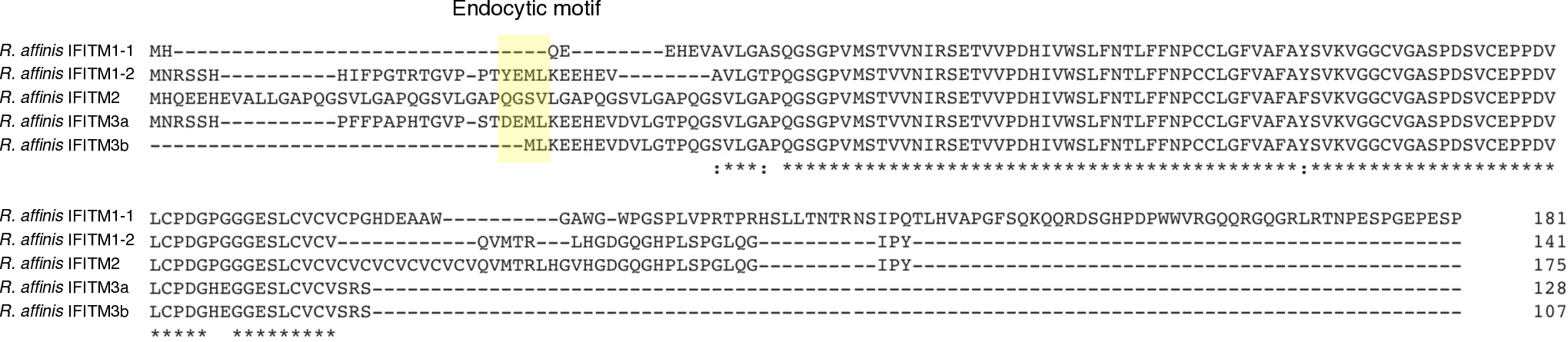

Supplement: S10 Fig — Protein sequence alignment of R. affinis IFITMs that show highest homology with human IFITM1-3. Location of the endocytic motif is highlighted. Asterisks (*) indicate positions with a conserved residue; colons (:) and periods (.) indicate conservation between groups of strongly and weakly similar properties respectively. (TIF) [file ppat.1012763.s010.tif]

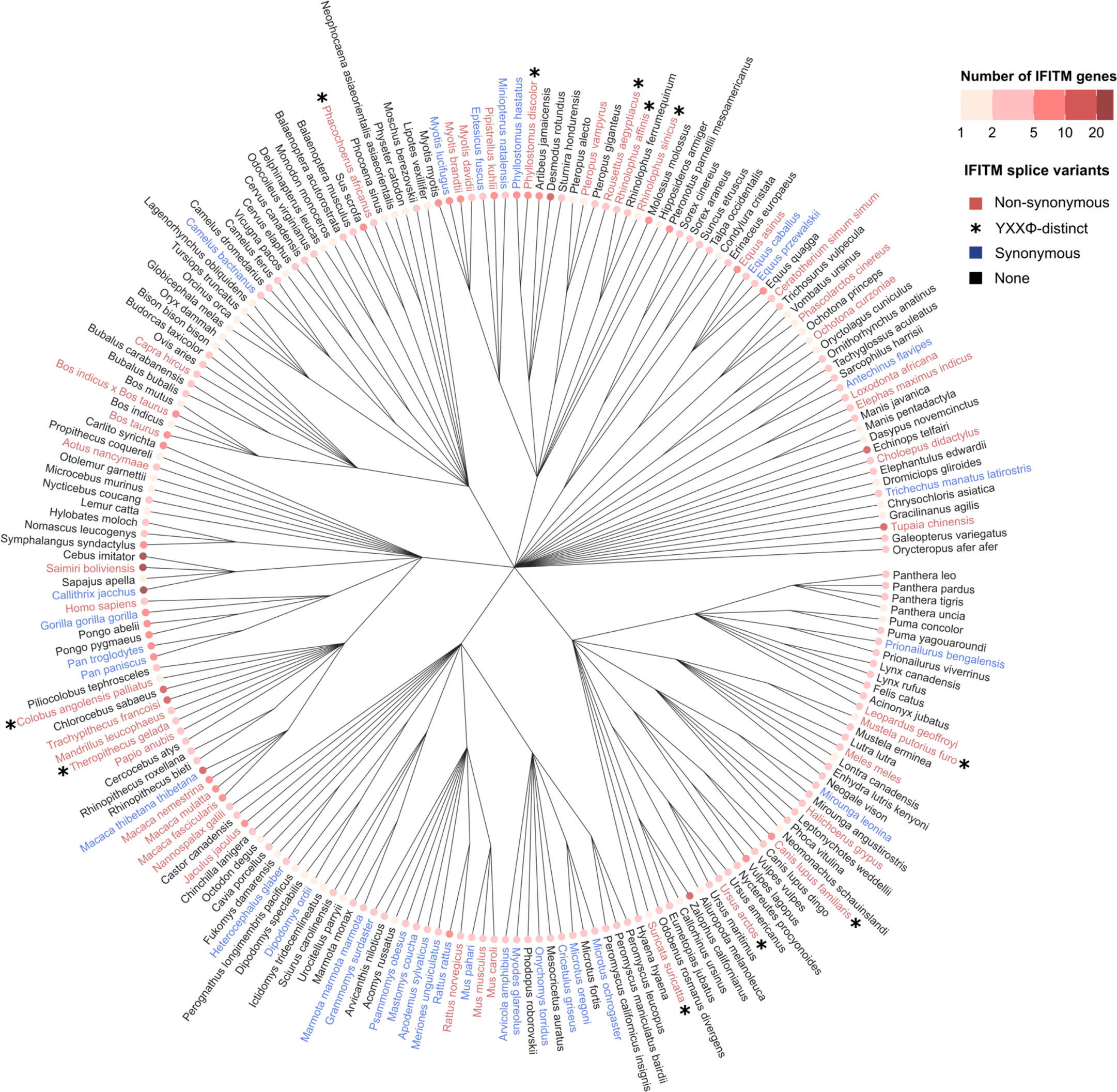

Supplement: S11 Fig — Analysis of IFITM-like genes in Fig 7A is extended to include 206 mammalian species. Mammals were grouped according to the IFITM-like genes they possess. Phylogenetic tree showing the ancestral relationships between these species was labeled by their grouping: species with IFITM-like gene(s) that encode two or more synonymous (blue) or non-synonymous (red) IFITMs are colored. Species with IFITM-like gene(s) encoding YXXΦ-distinct IFITM isoforms are marked with an asterisk (*). Tip nodes are colored by the number of IFITM-like genes they possess. (TIF) [file ppat.1012763.s011.tif]
