# Supplementary material for: Alternative splicing expands the antiviral IFITM repertoire in Chinese rufous horseshoe bats
Source: PLoS Pathog. 2024 Dec 26;20(12):e1012763. doi: 10.1371/journal.ppat.1012763 (PMC11801718; doi:10.1371/journal.ppat.1012763)
Supplement: Attachment — Submitted filename: Rebuttal_letter_20230910.docx [file ppat.1012763.s015.docx]

**Response to review comments and description of changes in manuscript**

**Description of changes**

We would like to thank the editor and reviewers for their constructive suggestions which has led to work that we feel have significantly enriched the manuscript. In line with the reviewers’ comments, we repeated some experiments to include more conditions and performed additional experiments which are summarized here:

1. Confirmed the expression of endogenous rsIFITMs in RsKT.01 cells by RT-qPCR and western blotting
2. Addressed the caveat of rsIFITM overexpression by comparing expression levels in transfected versus native cells, and measuring antiviral activity at different expression levels
3. Tested the effect of rsIFITMs on IAV at higher MOIs and more time points, and by negative imprinting
4. Measured the antiviral activity of rsIFITMs against two additional viruses – SARS-CoV-2 (3 VOCs) and Nipah virus pseudotypes
5. Tested the antiviral potencies of N-terminal rsIFITM mutants with altered localization

These experiments not only strengthened our conclusions, but also indicate a role of localization on rsIFITM antiviral activity which we confirmed with different experimental approaches. In additional to experimental additions, we also made writing edits in response to specific reviewer comments as detailed below.

While revising our manuscript, we noticed an error in the naming of transcripts in our RNA-seq plots (Fig. 1B-D). This error does not affect the conclusions in our revised manuscript and the figures have been corrected. All changes made to the manuscript are highlighted.

**Editor comments**

Additionally and in line with the respective reviewers' comments, please improve accuracy of the taxonomic description of bats and viruses of the study across the manuscript. For instance, please consider  "many Chinese horseshoe bat species" instead  of "Chinese horseshoe bats" or alike in the Title and elsewhere.

Thank you for this suggestion. “Chinese horseshoe bat” in our initial manuscript refers specifically to the species *Rhinolophus sinicus*, whose common name is “Chinese rufous horseshoe bat” in full. This has been changed in the title and throughout the manuscript (Lines 2, 37, 61, 391, 433, 450, 460, 467).

Likewise, consider "species *Severe acute respiratory syndrome-related coronavirus*" instead of "SARS coronaviruses" in references to the respective viruses infecting one or more bat species in the field. Consult <https://doi.org/10.1038/s41564-020-0695-z> for guidance on the nomenclature of coronavirus taxa that include SARS-CoV and SARS-CoV-2.

We agree that “severe acute respiratory syndrome-related coronaviruses” is a more accurate nomenclature and have made relevant edits in the manuscript (Lines 38, 62, 116, 117, 153).

**Major issues**

**Reviewer 1**

1) Fig 3: Different MOIs may lead to different outcomes. Testing the robustness of rsIFITM antiviral activity at different MOIs should be considered. The corresponding flow data (Fig. S3) shows that the authors claims are being made based on a small population of infected cells (~10% in control cells), so I question whether this phenotype is abolished at slightly higher MOIs. It would also be worth evaluating the kinetics of virus spread -/+ rsIFITMs (e.g., growth curve experiments with replication competent viruses) rather than single stagnant timepoints.

Reviewer 1 is correct to note that it is important to ensure results are robust to various infection conditions. To address this, we infected cells expressing rsIFITMs with influenza A virus at 3 MOIs, achieving 88% infection at the highest MOI and observing consistent results at all MOIs (S5B Fig, lines 237-239). Additionally, the kinetics of virus spread in the presence of rsIFITMs was assessed by measuring infection at 4 different time points (S5C Fig, lines 237-239). The results show that the distinct antiviral phenotype of rsIFITMa and rsIFITMb is robust and maintained at all MOIs and time points tested.

2) Fig 3: The authors should demonstrate how the magnitude of rsIFITM expression in transfected cells relates to endogenous expression in bat cell lines. For instance, in the rsIFITM transfected cells, is the mRNA abundance anywhere close to what was observed following ISG induction in bat cell lines? The authors should also consider transfecting cells with varying amounts of plasmid DNA and measuring dose effects on virus infectivity. Exceedingly high levels of overexpressed IFITMs may not accurately reflect the true natural state of the protein.

We agree that overexpression of exogenous proteins can be problematic to interpret. To compare IFITM expression in transfected HEK293T cells and endogenous expression in RsKT.01 cells, we performed absolute quantification by RT-qPCR as suggested by reviewer 1 (S2A Fig, lines 228-230). This shows that the transfection dosage used for most experiments (300 ng) resulted in IFITM expression levels comparable to that in RsKT.01 cells upon poly(I:C) treatment. In line with reviewer 1’s comment, we also transfected cells with various amounts of plasmid DNA (30, 100 and 300 ng), and we observed that rsIFITMs significantly inhibited HCoV-229E pseudotypes at all transfection dosages (S7A-B Fig, lines 249-251). Importantly, 100 ng transfections which resulted in lower-than-basal expression levels according to RT-qPCR measurements conferred strong antiviral activity.

3) Fig 3G: It was a nice addition to perform experiments in a relevant bat cell line. However, this is still quite artificial as the authors are relying on exogenous overexpression of rsIFITMs, and their phenotypes for 229E are still quite modest. To make claims that they are “antiviral in their native cell background” the authors would need to either knockdown (or knockout) these isoforms in bat cells and then assess virus infection. Even more important would be to determine whether knockdown (or knockout) in the presence of ISG induction (e.g., interferon stimulation) has any effects on infection outcomes.

This is a valid suggestion as endogenous and overexpressed IFITMs may exhibit different phenotypes, which we would very much like to address. The modest antiviral phenotype of overexpressed rsIFITMs in wild-type RsKT.01 cells is likely due to the high constitutive expression of rsIFITMa and rsIFITMb, as demonstrated by RNA-seq and RT-qPCR (Fig 1D, S2A Fig, lines 182-186). Knockout/knockdown experiments in native bat cells (RsKT.01) has however been technically challenging due to their slow growth, transfection/transduction refractoriness and the lack of good reagents for validation. We were able to successfully knockout rsIFITMa in RsKT.01 cells after multiple attempts, which confirmed its native expression (S2D Fig, lines 191-192). Unfortunately, we were unable to maintain the knockout cells in culture and therefore could not perform further infection experiments using these cells.

4) Fig. 4 The analyses of “FLAG signal on the cell surface” lack robustness. Line 888-889 “averages from 3 cells” is insufficient. The authors need to increase their cell number by imaging a set number of random fields of view. Using a membrane marker stain would strengthen this analysis. Quantification of CD63-FLAG colocalization would also be useful. As a caveat, it’s important to note that artificial overexpression may change the localization of IFITM proteins.

This is a fair point as single cell data may be particularly noisy, so it is reasonable to work with larger sample sizes in this analysis. Analysis was performed on additional cells (>30 cells per condition in total) expressing rsIFITMa/b by imaging random fields of views, and conclusions were unchanged (Fig. 4B, lines 280-283). Quantification of CD63/FLAG colocalization, as suggested by reviewer 1, further supported our conclusions (Fig. 4C, lines 283-284). We tried to incorporate a membrane dye (Membrite Fix) but it was technically challenging to limit staining to only the plasma membrane, so transmission images (pseudo-brightfield) were taken to improve the original analysis (tracing cell boundaries). We acknowledge the caveats surrounding with the overexpression in localization studies, this was minimized by ensuring comparable expression of transfected and endogenous rsIFITMs (S2A Fig, lines 228-230).

5) A western blot accompanying Fig. 6C would be important to show that differences in expression are not accounting for the different phenotypes observed. Also, given the modest phenotypes (<2 fold reduction at most), testing another virus isolate (or pseudovirus) would help add to the claim of “distinct antiviral specificities” between the different isoforms.

Infections in Fig. 3H and 7C (previously Fig. 6C) were performed in RsKT.01 cells stably expressing *R. sinicus* or *R. affinis* IFITMs. Since the IFITM constructs used co-expressed mCherry, cells were sorted for mCherry fluorescence during cell line generation to ensure that differences in expression do not account for the different phenotypes observed (Lines 541-543). The modest phenotypes could be explained by the high constitutive expression of IFITMs in RsKT.01 cells, which we were unfortunately unable to knockout as described above.

**Reviewer 2**

1. Please provide more details on RNA sequencing. The authors acknowledge that ‘absolute quantification of expression was not possible due to biased handling of reads which mapped to sequences shared by both isoforms’. How were the reads unambiguously assigned to one or the other isoform then (Fig. 1B, C, D)?

We apologize for the lack of detail in our explanation regarding RNAseq data analysis. Relative expression of rsIFITMa and rsIFITMb were calculated from reads that mapped to isoform-specific regions. Non-isoform specific reads were excluded from the differential isoform expression analysis. The methods section has been updated accordingly (Lines 483-488). Additionally, absolute quantification of endogenous rsIFITMa and rsIFITMb expression in RsKT.01 cells was performed by qRT-PCR to confirm the results of RNA-sequencing (S2A Fig, lines 185-186).

2. There is no evidence provided that proteins are indeed being generated and stably expressed from both native transcripts. All structural data are based on predictions and on analysis of synthesised peptides corresponding to merely the amphipathic helices.

This is a fair critique as our initial submission only addressed native IFITM expression through transcriptomic data (Fig. 1B-D). To support rsIFITM expression and upregulation in a native background, we measured expression of rsIFITMa/b in RsKT.01 cells (*R. sinicus* kidney epithelial cells) by western blot and RT-qPCR (S2A-C Fig, lines 185-188). Induction of both isoforms was evident upon poly(I:C) stimulation. The band corresponding to rsIFITMa detected by western blot was abolished upon CRISPR/Cas9-mediated knockout with a gRNA targeting the first exon of *rsIFITMa*, further confirming its endogenous expression (S2D Fig, lines 188-192).

3. Figure 3: please provide raw flow cytometry data showing the gating strategy and the specificity of the anti-FLAG immunostaining

Thank you for the comment, we agree that it is informative to show the gating strategy for infection experiments. S4 Fig has been updated to include raw data and detailed strategy for FLAG gating in experiments that involves IFITM transfection (Fig. 3B & F). Similarly, flow cytometry data for IFITM expression in stably transduced cells and for IAV infection in these cells are included in S6 Fig.

4. Figure 4: the rationale to test the impact of the RsIFITMa and RsIFITMb in RsKT.01 cells which already express endogenous levels of both (as shown in Fig. 1) is unclear – the antiviral activity of the heterologously expressed proteins might be underestimated due to the contribution of the expression of endogenous proteins.

Reviewer 2 correctly points out the limitations of our approach in testing the antiviral activity of rsIFITMa and rsIFITMb in their native background. This approach was chosen due to the technical challenges of knockdown/knockout in RsKT.01 cells and we acknowledge that the phenotype of transfected rsIFITMs could be underestimated. Figure 3H nevertheless shows reduction in infection upon rsIFITMa or rsIFITMb overexpression, suggesting their strong antiviral phenotype. Despite our attempts to knockout endogenous rsIFITMs in response to this and other reviewer comments, we were unable to maintain knockout cells in culture for infection experiments (S2D Fig).

5. Why weren’ t lentiviral pseudoptypes with SARS-CoV-2 spike, ideally corresponding to different VOCs not included?

This is a valid suggestion as SARS-CoV-2 remains to be a clinically relevant virus circulating in the population. We tested the effect of rsIFITMs on SARS-CoV-2 using pseudotypes corresponding to Wuhan, Delta and Omicron variants. Results strongly support the distinct phenotypes of rsIFITMa and rsIFITMb (Fig. 3G, lines 254-261).

6. The second phenotype of IFITMs, which is to lower infectivity of de novo generated particles, seems to be ignored in this study. How do the bat isoforms behave in corresponding assays?

Reviewer 2 raises an important point; negative imprinting is an alternative antiviral phenotype of IFITMs in which IFITM incorporation into nascent virions reduces their infectivity. To address this point, Influenza A virus pseudotypes produced from cells expressing human IFITM3 (positive control), rsIFITMa or rsIFITMb were tested for infectivity after normalization. Results indicate that rsIFITMa reduces the infectivity of *de novo* generated particles (S5D Fig, lines 239-243).

**Minor issues**

**Reviewer 1**

In the introduction and discussion, the authors discuss gene duplication and alternative splicing as mechanisms for evolutionary innovation in response to viruses. It would be worthwhile for the authors to also address how adaptive evolution through positive selection has influenced IFITM functional diversity in Chiropterans.

We agree that positive selection is an important mechanism of adaptive evolution that has led to IFITM functional diversity in bats, whereas gene duplication and alternative splicing are strategies to resolve adaptive conflicts which would otherwise limit the rate of positive selection. This has been added to the introduction (lines 127-131). In addition, positive selection of bat IFITMs and variation at codon 70 have previously been reported. This is further elaborated in the discussion (lines 406-409).

Line 382-385: This is a major speculation based on the data presented. Only a limited set of viruses were tested (1 influenza and multiple CoVs), and it’s difficult to say that one is more antiviral than the other without a more representative (diverse viral families) set of viruses. It’s possible that each are specialized in their own unique ways. Perhaps consider toning down this language or discussing potential alternative theories.

Reviewer 1 correctly points out that rsIFITMa and rsIFITMb could be specialised in their own ways. To address this, we (1) tested an additional virus family (paramyxovirus) and (2) the antiviral activity of rsIFITM mutants with altered localizations (Fig. 5, lines 286-310), which led to a revised conclusion. These data strongly suggest the unique specialization of rsIFITMa and rsIFITMb against different virus families, as reviewer 1 suggested. We have removed our initial suggestion that one is “more antiviral” than the other and are instead proposing that they may be specialized to restrict viruses that use different routes of entry (lines 401-403, 409-412).

In Fig 3, the data is normalized based on vector transfected cells, where Fig S3 labels the control as “untransfected.” Which is correct here? Using untransfected cells as a control is not appropriate as the mere act of transfection may influence the infection. A more detailed gating strategy in S3 would be helpful to see how many cells were successfully transfected.

This comment identified an error in the manuscript and we would like to thank reviewer 1 for pointing this out. The labels in the original Supp. fig. S3 should be “vector-transfected”, instead of “untransfected”, and is corrected in the updated figure (S4A-B Fig). All infection levels were compared to that in vector-transfected control cells to account for the effect of transfection. Detailed gating strategy for all influenza A virus and HCoV-229E-GFP infections including the percentage of successfully transfected cells have been added to the manuscript (S4C Fig).

Fig 3 figure/legend:
(B) For clarity, the authors should note that IAV infection was assessed by NP staining. I had to dig into the supplement figure/methods to find this out. MOIs should also be noted.
(C-E) Similarly, that the lentiviral pseudotype vectors encode a luciferase transgene. It’s implied by the readout, but not explicitly stated in the legend.
(G) I’m confused by “infected with serially diluted.” Do you mean cells were infected and then cell supernatants were serially diluted to quantify infection by TCID50? This also comes up in Fig 6C. Please clarify.

All the suggested edits have been made to the legend (Lines 1046-1078). We would like to apologize for the lack of clarity in our initial description of HCoV-229E infections in RsKT.01 cells. Reviewer 1 is correct in that cells were infected and infectious virus titer in the supernatants were quantified by TCID50 assay. The methods section has been updated accordingly (Lines 573-577).

Fig 3. Please make the y-axis consistent for all figures. The y-axis for E and G are different than the others.

The y-axis scale for Fig. 3E has been edited to match the other graphs. As for Fig. 3H, the y-axis is slightly extended to ensure that the error bars are fully shown.

Fig 6A. Genus/species names should be italicized.

All genus and species names are now italicized (Fig. 7A).

Line 73: “Dengue” should be “dengue.”

The capitalization of “Dengue” has been corrected (Line 76).

Line 837: these plots don’t match up with their descriptions. “B” is VSV infected, and “C” is poly(I:C) treated, but the legend states “poly(I:C)-treated (B) or VSV-infected (D) cells.” Please correct either the figure labeling or legend.

This figure legend has been corrected to ensure the descriptions match with the figures (line 1025).

Lines 361-364: Two different points are being made. Please consider splitting the argument into two sentences.

The arguments have been split to improve the flow (lines 424-427).

**Reviewer 2**

1. Lines 31-32: ‘the unique IFITM repertoire in different species influence their tolerance to viral infections’: tolerance or resistance?

We agree that “resistance” is more accurate than “tolerance” and the replacement has been made (line 34).

2. Line 66 “dictates” change to ‘dictate’

This grammatical error has been fixed (line 69).

3. Line 72: ‘IFITM3 is the most well-studied’ change to ‘IFITM3 is the best-studied’

The suggested phrase has been used (line 75).

4. Lines 107, 475 and elsewhere: ‘SARS-CoV-1’ change to ‘SARS-CoV’

All relevant changes were made in the manuscript.

**Reviewer 3**

1. The rationale for the inability to perform absolute quantification of expression in line 171 and Figure 1 is unclear. Could real-time PCR, targeting each specific isoform, facilitate quantification without the need for RNAseq, given the focus on rsIFITM genes?

We apologize for the unclear explanation for the RNAseq data analysis and agree that qPCR-based quantification of rsIFITM expression would strengthen our manuscript. Relative expression of rsIFITMa and rsIFITMb were calculated from reads that mapped to isoform-specific regions. Non-isoform specific reads were excluded from the differential isoform expression analysis. The methods section has been updated accordingly (Lines 483-488). Additionally, absolute quantification of endogenous rsIFITMa and rsIFITMb expression in RsKT.01 cells was performed by qRT-PCR to confirm the results of RNA-sequencing (S2A Fig, lines 185-186). Protein expression of rsIFITMs was also detected by western blotting (S2B-C Fig, lines 186-188).

2. The Methods section requires elaboration. For instance, the origin or source of the RsKT.01 cell line, details on the transfection procedure, and normalization methods need clarification. In Figure 3A, a disparity in the expression levels of IFITMa and IFITMb proteins is observed; does this contribute to the differing antiviral effects between the isoforms? The stability and passage number of the cell lines used prior to infection should also be clarified. In general, the current description lacks sufficient detail for replication.

We have elaborated the methods section in response to these points: RsKT.01 cells are an immortalized *R. sinicus* kidney epithelial cell line (line 510, ref. 60); ratios and reagents used in transfection were included (lines 531-538); normalization against vector-transfected cells in infection experiments is explained (lines 577-579, 593-595). Additional details have also been added to other parts of the methods section to ensure reproducibility.

We acknowledge the disparity in rsIFITMa and rsIFITMb expression shown in Fig. 3 but argue that this does not explain their distinct antiviral activities. Newly added data shows that rsIFITMb remains less antiviral than rsIFITMa against IAV in stably transduced cells despite its higher expression level (S5A-C Fig, line 237-239). Differences in antiviral potency is also preserved at varying transfection dosages to a degree that a 10-fold lower expression of rsIFITMa still maintained a comparable antiviral activity as rsIFITMb (S7A-B Fig, lines 249-251). Moreover, Nipah virus entry is restricted to a greater extent by rsIFITMb than rsIFITMa, suggesting that disparity in expression level cannot explain the differing antiviral effects (Fig. 5D, lines 303-309).

In addition, we have noticed that apparent differences in expression level shown in western blots could be explained by differences in lysis efficiency. We further confirmed this by assessing IFITM expression in transfected HEK293T by flow cytometry (as shown in the figure here). Normalized mean fluorescence intensities for Flag staining supports the lack of significant difference between rsIFITMa and rsIFITMb expression.

3. In lines 229 and 295, the authors discuss the distinct antiviral specificities of rsIFITMa and rsIFITMb (and similarly for raIFITM3a and raIFITM3b), yet the presented data do not convincingly demonstrate this specificity. The study primarily shows that isoform a consistently exhibits stronger antiviral activity than isoform b across Figures 3, 4, and 6. Clarification on the definition of specificity versus activity is needed, especially since the study does not establish a correlation between IFITM localization and antiviral specificity (line 343).

Reviewer 3 reasonably challenges the conclusion of “distinct antiviral specificity” between rsIFITMa and rsIFITMb. We believe that the additional experiments included address this critique. For instance, we show that rsIFITMb restricts Nipah virus entry to a greater extent than rsIFITMa (Fig. 5D, lines 303-309). We also established an effect of IFITM localization on its antiviral specificity using N-terminal rsIFITM mutants which, we believe, strengthens the conclusion of our manuscript (Fig. 5A-C, lines 288-298). Data showing that rsIFITMb, but not rsIFITMa, enhances SARS-CoV-2 entry further supports the distinct functions of the two isoforms (Fig. 3G, lines 254-261).

4. The use of the TCID50 assay in Figures 3G and 6C raises questions. This assay does not follow the traditional approach of measuring virus produced in the supernatant. The rationale behind not titrating the virus in the supernatant should be explained. Additionally, the representation of TCID50 values typically employs a log scale, making the 60-80% infectivity relative to the vector control appear minimal. Details on the number of biological replicates, stable cell lines, and independent infections conducted would greatly benefit the reader. Additionally, why TCID50 was used instead of IFA to quantify the infectivity?

We would like to apologize for the misleading description of Fig. 3G and 6C (now Fig. 3H and 7C respectively) in the legends and methods section. In fact, RsKT.01 cells were infected with HCoV-229E and infectious virus titers in the supernatants were measured by TCID50 assay following the conventional approach. Infection was quantified by TCID50 because HCoV-229E replication takes place in replication foci in RsKT.01 cells which was not robustly detected by immunofluorescence staining. The corresponding sections have been updated to include a clear description of the experimental methods used (lines 575-577, 1071-1073, 1131-1133).

The modest inhibition by overexpressed rsIFITMs in RsKT.01 cells may be due to the high constitutive expression of rsIFITMa and rsIFITMb, as demonstrated by RNA-seq and RT-qPCR (Fig. 1D, S2A Fig, lines 181-186). Unfortunately, we were unable to perform infection experiments in knockout cells due to the technical challenges associated with the generation and maintenance of IFITM knockout RsKT.01 cells. We believe that Fig. 3G nevertheless supports our conclusions by showing the differential degree of virus inhibition by rsIFITM isoforms in their native cell background.
