# Supplementary material for: Alternative splicing expands the antiviral IFITM repertoire in Chinese rufous horseshoe bats
Source: PLoS Pathog. 2024 Dec 26;20(12):e1012763. doi: 10.1371/journal.ppat.1012763 (PMC11801718; doi:10.1371/journal.ppat.1012763)
Supplement: Attachment — Submitted filename: Rebuttal letter_third submission_20241106.docx [file ppat.1012763.s016.docx]

**Response to review comments and description of changes in the manuscript**

**Description of changes**

We would like to thank the editor and reviewers for their constructive suggestions throughout the review process. In response to the review comments, we made writing edits to improve the accuracy of our wordings and expanded on the methods section as detailed below. We also deposited all code scripts for the bioinformatic analysis on Github for reproducibility.

In line with the figure requirements, we reformatted our main figures to fit publication standard, but their contents remain unchanged.

**Part I – Summary**

Reviewer #1: I appreciate the effort put in to addressing this reviewers’ comments. I believe the manuscript has been improved by the additional experiments and corrections made throughout. However, I do have one concern that I would like the authors to address.

Reviewer #2: The authors have addressed my concerns satisfactorily.
-They should please add the source and/or information regarding the production of IAV, VSV and HCoV-229E-GFP, and plaque/TCID50 assays.

Thank you for the kind reminder, all relevant information has been added to the methods section: IAV (lines 566-569), VSV (lines 477-478), HCoV-229E (lines 570, 579-580) and TCID50 assay (lines 580-589).

-lines 485/486: "at a 1:3 molar ratio of plasmid DNA to transfection reagent", this does not make sense because DNA is in ug and Fugene in ul, so it is not a molar ratio.

Thank you for pointing this out, this is now corrected (line 522).

-throughout the paper: the correct term is "immunostained" (for antibodies), not "stained"

All relevant wordings have been changed (lines 232, 572, 576 etc.).

-throughout the paper: "SARS-CoV and MERS-CoV pseudotypes" please change to "SARS-CoV spike and MERS-CoV spike pseudotypes". The pseudotypes are lenti/retroviral based and contain merely the heterologous glycoprotein/spike/HA of indicated viruses.

We agree that it is important to make the distinction between authentic and pseudotyped viruses. For that reason, we stated clearly that these pseudotypes are lentiviral-based and only express the surface glycoproteins of the virus of interest (lines 248-249). In line with reviewer 2’s comment, we changed the naming into “CoV spike pseudotypes” in the first instance (line 250). These pseudotypes are commonly termed “CoV pseudotypes” in other published papers and we therefore did not modify the naming of following occurrences (e.g. Cantoni et al. (2022) Nature doi: <https://doi.org/10.1038/s42003-022-03325-9>).

Reviewer #4: In this study, Mak et al. explore the IFITM repertoire of Chinese rufous horseshoe bats and its antiviral properties against several human pathogenic zoonotic viruses, such as SARS-CoV2, IAV and Nipah virus. Using a range of molecular techniques, they show that this bat species encodes two IFITM isoforms as a result of alternative splicing and show that the two isoforms exhibit distinct cellular localization and different antiviral properties against various pseudoviruses and replication-competent viruses used in the study. After the first round of revisions, the authors have addressed overall well the first set of reviewer questions and comments, and notable improvements in the manuscript and dataset are evident. However, I do have several additional questions or comments on the text and figures.

**Part II – Major issues**

Reviewer #1: In response to this reviewers’ comments, the authors performed additional studies described in Supplementary Figure 5. While rsIFITMa consistently shows a restrictive phenotype, the data presented for rsIFITMb now conflicts with that presented in Fig. 3. See revised manuscript lines 213-227. rsIFITMb is described as inhibitory in Fig. 3B (statistically different from vector transfected cells), yet the percent cells infected are on par with that of the vector control in Fig S5B/C (i.e., rsIFITMb is not inhibitory). These are two contradictory phenotypes that warrant discussion, as it potentially calls into question the modest restrictive phenotypes of rsIFITMb for other viruses in Fig. 3 and reproducibility amongst experimental replicates.

Reviewer 1 raised a valid point that we agree should be addressed in the manuscript. IAV PR8 infection experiments included in the initial submission (Fig. 3) were performed by transiently transfecting cells with IFITMs and infecting them at 48 hours post-transfection. On the other hand, additional experiments (Fig. S5) were performed in cells that were stably transduced with IFITMs. Since we had to perform infection experiments at various MOIs and time points during revision, we decided to generate stable cell lines to minimize variations arising from transfection. The different expression systems (transfection vs transduction) could explain the observed difference in rsIFITMb antiviral potency against IAV. Residual responses to the transfection reagent and plasmid DNA at the time of infection may contribute to the inhibition seen in Fig. 3. Differences in IFITM expression levels in these two expression systems may also play a role. This discrepancy is now addressed in the manuscript (lines 240-242).

Most importantly, the conclusion we draw from these experiments is sustained – the differential antiviral potencies of rsIFITMa and rsIFITMb. Fig. S5 provides additional support that this differential phenotype is robust and can be observed at various experimental conditions.

Reviewer #2: (No Response)

Reviewer #4: Lines 111-112: “Constitutive expression of IFNs in bats” is a somewhat incorrect statement, due to how broad the statement itself is regarding “bats”. Constitutive expression of IFN has been shown in only a few bat species so far, with emerging evidence across other species showing no evidence of constitutive IFN expression. Therefore, I’d suggest altering this statement to reflect that constitutive IFN expression isn’t actually a Chiroptera-wide phenomenon. It's a feature of some bat species, but not others.

Reviewer 4 correctly suggested that constitutive IFN expression may not be true for all bat species considering the huge diversity within the order Chiroptera. While the type of IFNs and the extent of their constitutive expression are less clear, constitutive expression of ISGs have been consistently observed in various bat species. To reflect this, the wording of the statement was modified and relevant references were added (lines 117-119).

Lines 561-579 (Replication-competent virus infection) and Supplementary Figure 4: Was a dead cell exclusion dye included in the flow cytometry analysis? There is no mention of a live/dead differentiation, which is a crucial first step in any flow cytometry approach. Dead cells are highly autofluorescent in most channels, so failing to exclude them during gating can significantly distort population percentages and overall end results. Not having performed dead cell exclusion would be a big flaw in the experimental design of the flow cytometry data in Fig. S4. Same comment applies to the gating strategy in Fig. S6.

We appreciate this suggestion from reviewer 4. While including a live/dead stain is a valid strategy for excluding dead cells, we feel that our approach sufficiently allows live/dead cell differentiation. Our flow cytometry-based analysis of infection follows the methodology of published studies (e.g. Chesarino *et al.* (2017) EMBO reports doi: [10.15252/embr.201744100](https://doi.org/10.15252/embr.201744100); Rahman *et al.* (2022) JMB doi: [10.1016/j.jmb.2022.167759](https://doi.org/10.1016/j.jmb.2022.167759)).

Firstly, infected cells were closely monitored post-infection and minimal cytotoxicity was observed at the infection end-points. Secondly, any floating cells were removed by washing the adherent cell layer with PBS prior to fixing and immunostaining. Thirdly, FSC/SSC gating was included as the first step in the analysis pipeline of all flow cytometry data. These steps ensure that dead cells are excluded from the data being analyzed.

**Part III – Minor issues**

Reviewer #1: (No Response)

Reviewer #2: (No Response)

Reviewer #4: Line 416: Minor vocabulary correction - “with horseshoe bats as an exemplar” should be corrected to “with horseshoe bats as an example”.

This has been corrected (line 418).

Perhaps I’ve missed it, but I didn’t see an explanation in the Methods where the RsKT.01 cell line originated, was gifted or was purchased from.

The RsKT.01 cell line was previously developed by us and used in previously published studies. This has been made clear with an added reference in the methods section (lines 512-513).

For all the bioinformatics analysis performed in this study, I strongly suggest that the authors make the code scripts they used available on a repository such as GitHub or any other alternative, in the name of transparency and reproducibility.

Thank you for this suggestion. We have created a github repository containing all code scripts for the analysis of alternative splicing in mammalian IFITMs. This link to this repository is included under the data availability and methods section (lines 657-662).

Lines 493-499: Please include the dilutions of each antibody.

All antibody dilutions used have been added (lines 530-537).
